# Port of Spain Summit Declaration as a successful outcome of global health diplomacy in the Caribbean region: a systematic review

**DOI:** 10.15171/hpp.2019.25

**Published:** 2019-08-06

**Authors:** Vijay Kumar Chattu, Andy W Knight

**Affiliations:** ^1^Faculty of Medicine, University of Toronto, Toronto, Victoria St. ON M5B 1T8, Canada; ^2^Institute of International Relations, The University of the West Indies, St. Augustine, Trinidad and Tobago; ^3^Department of Political Science, Faculty of Arts, University of Alberta, Canada

**Keywords:** Global health, Diplomacy, Non-communicable Diseases, Epidemics, Caribbean region, Disease prevention

## Abstract

**Background:** The Caribbean region, with a population of around 17 million, has the highest burden of chronic noncommunicable diseases (NCDs) in the region of the Americas. It is estimated that diabetes and hypertension has an economic impact of around 5%-8% of the gross domestic product of the region. The purpose of this study was to explore and understand how global health diplomacy contributed to the evolution of a collective Caribbean regional summit declaration to address the epidemic of NCDs.

**Methods:** A systematic review was conducted, and all the major databases such as MEDLINE, PubMed, EMBASE, Scopus, Web of Science, EBSCO, Global Health database and other available policy documents from various sources were screened. All articles available from 1910-2018were extracted. From the total of 3223 titles, after filtering, the search narrowed down to 28full texts that are included in this study. Policy documents and articles related to NCDs, global health diplomacy, and the Port of Spain Declaration (POSD) were the focused themes.

**Results: ** The Caribbean region showed significant commitment to the prevention and control of NCDs through its united voice and commitment since 2001. The successful rounds of negotiations for regional health have led to the formulation of the 15- point multisectoral POSD "Uniting to Stop the Epidemic of Chronic NCDs." This was the first Summit in the world where the Heads of Government focused on prevention and control of NCDs with a clear road map for policy implementation, collaboration, and collective action. This regional summit declaration gained global attention and resulted in the United Nations Political Declaration on the Prevention and Control of NCDs and as WHO Global Action Plan for the Prevention and control of NCDs 2013-2020.

**Conclusion: ** There is enormous scope for this evolving area of Global Health Diplomacy in addressing the future challenges of health security.

## Background

The Caribbean Community (CARICOM) comprises of 15 member states and five associate members which has a population around 17 million has the highest burden of chronic noncommunicable diseases (NCDs) in the region of the Americas. The region contributes to 30% of all deaths due to the preventable NCDs that occur prematurely.^1^ To highlight some cases, diabetes-related lower extremity amputations in Barbados are among the highest recorded in the world. The diabetes-related mortality is 600% higher, and cardiovascular diseases mortality is 84% higher in Trinidad and Tobago compared with North America. It was estimated that the economic impact caused by diabetes and hypertension contributes around 5%-8% of the gross domestic product in the CARICOM region.^2^ The epidemic of NCDs is exploding in the region especially among the poor and middle class due to their exposure to habits like alcoholism, tobacco use, unhealthy diets and lack of proper access to preventive and curative services which leads to premature deaths and push the families into poverty.^3^ Global health diplomacy (GHD) is an emerging interdisciplinary concept and was defined in different ways by various experts with different theoretical and disciplinary backgrounds, but there is no common definition agreed by all. According to Kickbusch et al,^4^ GHD is defined as “multi-level, multi-actor negotiation process that shape and manage the global policy environment for health.” The study aims to explore and understand how GHD contributed to the evolution of a collective Caribbean regional summit declaration in tackling the rising epidemic of NCDs.

## Materials and Methods

### Information sources and search strategy

The following keywords were used to search: Non-Communicable Diseases, Global Health Diplomacy, and Health Security, and the results were filtered by searching “Port of Spain Summit Declaration.” The following major databases were searched: MEDLINE, PubMed, EMBASE, Scopus, Web of Science, EBSCO and Global Health database. All articles available from 1950 to 2019 were extracted and review of policy documents related to GHD, NCDs was done. To fulfil the study objectives, a detailed review of policy documents, published research on NCDs in Caribbean region, Summit declarations, CARICOM meetings, Caribbean regional conferences of PAHO/WHO, Inter-American Development Bank were considered for this review. Databases were further screened for articles on “Port of Spain Summit Declaration,” “Global Health Diplomacy and Caribbean” and “Non-communicable diseases and Health policies in the Caribbean.” The important websites of CARICOM secretariat, Caribbean Public Health Agency (CARPHA), Pan American Health Organization (PAHO)/WHO, Government of Trinidad and Tobago and Institute of International Relations at University of the West Indies were searched to gather additional information on the topic of interest and were scrutinized for the required keywords and content.

### Inclusion and exclusion criteria

We included all the research articles published in the English language from 1910 till December 2018 as retrieved by the databases. Only articles in English language and with full texts were included in the study. The exclusion criteria for our study were the following: abstracts, articles other than English language, duplicates, articles covering other geographical regions other than the Caribbean region and articles without explicit methodology or results

### Study selection and data extraction

Two authors (VC and AK) independently assessed the study eligibility by reviewing the titles of all potential citations. Discrepancies were resolved by consensus between the reviewers. Full texts of all the relevant articles were assessed, and the data were extracted from each eligible study on the location of the study, author’s name and the presence and discussion of the keywords of interest. The articles on NCDs, health diplomacy, and health security, giving more relevant details about the Caribbean region were chosen.

## Results

**Figure 1:**
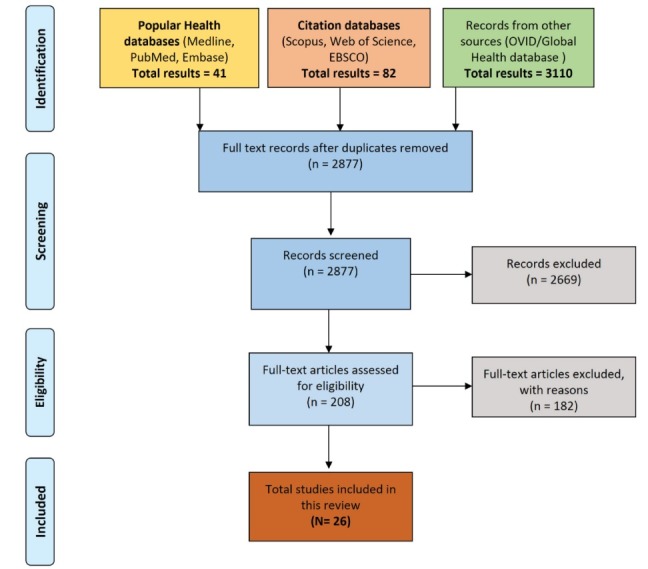
Flow diagram showing the literature search strategy.

The initial search on Ovid/Global Health databases for ‘Non-Communicable Diseases’ yielded 3110, including abstracts and full texts. After assessing the title and abstracts, only full texts were considered, and they are further filtered for “Health Security” and “Global Health Diplomacy” which yielded a total of 208 results which were further narrowed down by filtering with “Port of Spain Declaration.” A total of 28 full texts were retrieved for more detailed evaluation, and they were included in the current study. Figure 1 shows the results of the search strategy according to the PRISMA (Preferred Reporting Items for Systematic Review and Meta-Analysis) flow diagram.

All the major policies and significant findings from the regional meetings were identified and shown in tabular form with a time sequence and discussed below.

### Response of CARICOM

The first International Conference on Health Promotion took place in 1986, The Ottawa Charter for Health Promotion pledged to advocate a clear commitment to health, counteract unhealthy living conditions, to focus on public health issues and to reorient health services towards promotion of health.^5^ The charter also emphasized to recognize health and its maintenance as a social investment and challenge which was taken into serious consideration by the Caribbean region to implement in the region. Though the NCDs emerged as a major economic and human burden in many low-middle income countries and often neglected by the governments with a persistent myth that NCDs are the problem of developed nations, the region was determined to curb the rising epidemic. The Caribbean region has a great history of success for being the first region in eliminating indigenous polio, measles, rubella, and its response to HIV/AIDS.^6,7^ The region is a pioneer in developing policies for the prevention and control of NCDs as it showed its united voice and commitment in 2001 during Nassau conference. It also established Caribbean Commission on Health and Development “to propel health to the center of the development agenda” chaired by George Alleyene, the former director of PAHO/ WHO and former Chancellor of the University of the West Indies. During 2005-2006, the report of Caribbean Commission on Health and Development was helpful in advocacy activities to prioritize the NCDs and also for a regional summit of CARICOM Heads of Government on Chronic NCDs in Port of Spain, Trinidad and Tobago in September 2007 with the support from CARICOM, PAHO/WHO, Public Health Agency of Canada (PHAC). The successful GHD has led to the formulation of the 15- point multisectoral Port of Spain Summit Declaration “Uniting to Stop the Epidemic of Chronic NCDs.” Moreover this summit was backed by PAHO/WHO to provide the technical support for Surveillance, mobilizing other partners and resources, developing NCD plan and implementation.^8^ This summit was the first in the world where the Heads of Government focused on prevention and control of NCDs with a clear road map for policy implementation, intersectoral collaboration, and a collective action. The summit involved diverse actors from international organizations, the private sector, civil societies, and supporting governments.

### Global Health Diplomacy and evolution of the “Port of Spain Summit Declaration”

Early in 1986, the Caribbean Cooperation in Health Initiative was approved by the health ministers and made NCDs a priority to focus.^9^ This collaboration continued in the region since then with regular regional meets until it resulted in the formulation of the broader Caribbean centric policy for prevention and control of NCDs. GHD intersects the areas of health, foreign policy, and trade. Because of the rising threat of NCDs in the region, there is a great need and demand for GHD as the ministries of health or head of the states need to work to promote the health of their citizens along with the global community. Moreover, because of the growing burden of NCDs in the region, the regular meetings between foreign ministers and health ministers have become more common and frequent to address the critical aspects of population health.^10^ Health domain has taken a special position in the international relations, and health is used for soft power, developing security policies, trade agreements since it touches the issues of national development and economic interests. A summary of NCDs specific landmark events in the Caribbean region, as shown below (Table 1).

After a few rounds of brainstorming, debates, and negotiations, it was felt that there is a great need to address this epidemic comprehensively with upstream multisectoral policies and downstream health sector focused activities addressing the NCDs. The Port of Spain declaration (POSD ) is a result of long negotiations and successful health diplomacy with a high-level political commitment. The Summit declaration is a 15-point multisectoral declaration addressing the broader and core issues of tobacco, alcohol, healthy diet, physical activity, health services, monitoring, mobilizing society, and celebrating annual Caribbean Wellness days.

## Discussion

### Impact of “Port of Spain Summit Declaration” at regional level

Trinidad and Tobago have passed robust tobacco legislation, which became the model for the region if they chose to. As a result of this declaration, PAHO/WHO has developed an NCD surveillance (a model web-based reporting) with the support from Inter-American Development Bank. The summit also resulted in the establishment of a Healthy Caribbean Coalition which is a 30-member civil society alliance as a direct response to the NCD summit declaration.^7^ As the countries started implementing this regional declaration, the progress is monitored and tracked using a reporting grid that is submitted annually. Smaller countries have appointed one officer assigned to NCDs with other portfolios whereas larger countries have set up NCD department. It was found that due to the regional/global support for addressing the capacity as part of the declaration, programs such as Caribbean Wellness day, Framework Convention on Tobacco Control ratification, Global School Health, Global Youth Tobacco and STEPS NCD risk factor surveys have been implemented in all countries irrespective of size and capacity.^11^ Barbados has also inaugurated an ‘Inter-Ministerial Task Force on NCDs’ chaired by the Minister of Health and tasked to oversee the ‘whole of government’ response to NCDs.^12^ The Caribbean with its cultural diversity and high burden of NCDs provides an ideal environment within which to undertake further studies to better understand the interactions between culture and health policy formation.^13^

### CARICOM summit as a precursor of UN high-level meeting on NCDs at global level

The legacy and journey of this regional summit to the global level is shown below (Table 2). This summit declaration is a very good example of a successful summit as the follow up took place within a month of the summit and repeatedly afterwards with various stakeholders.^11^ In 1997, to prepare a regional strategy and plan of action numerous consultations with 35 countries and academic institutions were done to form the CARMEN (Collaborative Action for Risk Factor Prevention and Effective Management of NCD Network), for the integrated prevention and control of chronic diseases in the Americas and to ensure ownership by these countries.^14^

The initiative to produce POSD had begun with three members of the Caribbean Commission on Health and Development, some of whom sought a CARICOM summit as a pathway to reach the United Nations.^15^ Essential financial support came from outside the Caribbean region, specifically from Canada. The 2007 POSS planners focused the agenda on prevention, leading to CARICOM commitments on physical activity, nutrition, healthy diets, smoking reduction, preventive treatment and education, surveillance, monitoring, and evaluation.^16^ In October 2005, a reorganization of the structure of the PAHO/WHO was implemented in the Caribbean region by forming a new PAHO Office of Caribbean Program Coordination (OCPC) which is the coordinating entity for technical cooperation strategies at the Caribbean regional level. The OCPC is the assigned PAHO/WHO technical cooperation entity for the Caribbean Cooperation in Health, the blueprint for the strategic health agenda for the CARICOM region.^17^ In 2011, Kirton et al, from the Global Health Diplomacy program at the University of Toronto had put forward ten measures even before the UN summit to help to ensure a grand success. They include Multi-stakeholder Engagement; Development, Economic and Broader Health Links; Leader Involvement; Leadership Roles; Outcome Document; Implementation; Supporting Summits and HLMs; Funding; Accountability; Follow-up of UN HLM on NCDs.^18^ The NCDs impact as a development challenge was highlighted by Knight et al, in their report “Keeping NCDs as a Political Priority in the Caribbean: A Political Economy Analysis of Non-Communicable Disease Policy-making.” The report underscored that the NCDs negatively affect economies, health systems, households, and individuals through a range of drivers such as reduced labor productivity, higher medical treatment costs, absenteeism, and lost savings. These drivers aggregate into significant socioeconomic negative impacts, including in the areas of country productivity, competitiveness and fiscal pressures; ultimately undermining sustainable development goals.^19^

The advocacy by Sir George Alleyne at different global platforms^20^ and others resulted in first in the world Heads of Government NCDs Summit. This Summit Declaration served not only as a rallying point to accelerate the regional NCDs response, but also as a catalyst for the first United Nations high-level meeting on NCDs in September 2011.^21^ Kirton et al have stated that the CARICOM members had global first mover advantage in pioneering the world’s first summit on NCDs at Port of Spain in 2007.^22^ As highlighted in the Inter-American Development Bank report by Milano, the Caribbean seeks to create a common language and to share information, promote constructive dialogue, improve consultations, collaborations and partnerships among Governments, Private Sector and Civil Society.^23^ For the first time in the world, all the heads of government exclusively focused on NCDs resulting in multiple collective, multilateral commitments for the implementation of policies and actions about NCD control. It has sparked the interest in the global summit and was elevated to a global level and resulted in UN-High-Level Meeting on NCDs for the prevention and control of non-communicable diseases declared by the UN in September, 2011.^24^ Therefore GHD is now conducted in multilateral contexts as a method of reaching compromise and consensus in matters related to health so that new agreements promoting values and principles in the face of health threats (e.g NCDs) are adopted. the practitioners and professionals of both global health and foreign policy should work together with better understanding and coordination in order to improve global health security.^25^ Therefore GHD has the great potential to promote peace and security thus a complete understanding of the reasons for this, and the scope for furthering this perspective, remains a priority.^26^

## Conclusion

The Caribbean region has collectively identified the emerging epidemic of NCDs as a top priority and jointly committed with a strong political will to stop the epidemic. The immediate follow-up of the summit declaration of Port of Spain by engaging all the stakeholders in its implementation is a major highlight of this declaration, which has successfully attracted the global attention within a short time frame. The critical role of GHD is highlighted again through this declaration by becoming a global policy approved by the UNGA and WHO resulting in the WHO Global Action Plan for the Prevention and Control of NCDs 2013-2020 as resolution WHA66.10. The Port of Spain Summit Declaration is a very good example of a bottom-up policy with the involvement of all the stakeholders from the roots, which resulted in global policy. There is a great scope for this evolving area of GHD in addressing the future challenges of a range of health-related issues and health threats including emerging infectious diseases, trade in health, medicines, vaccines apart from epidemics.

## Ethical approval

The review is part of doctoral research by the first author (VC) under the supervision of AK, which was approved by the Campus Ethics Committee at The University of the West Indies, Trinidad, and Tobago (CEC723/09/18). This research was conducted at the University of Alberta, Edmonton, Canada as part of short-term visiting scholar at China Institute.

## Competing interests

The authors declare that they have no competing interests.

## Funding

The study did not receive any funding.

## Authors’ contributions

VC did the conceptualization, design, and initial draft. AK supervised the development of work, provided critical comments and did the manuscript evaluation. Both VC and AK approved the final draft.

## Acknowledgments

The authors would like to thank Prof. Gordon Houlden, Director of China Institute, University of Alberta for his cooperation and support.

**Table 1:** NCDs specific landmark events in the CARICOM since 1986-2011

**Year**	**Regional Forum/Cooperation**	**Main Outcome**
1986	Caribbean Cooperation in Health initiative	Approved by ministers of health
1995-2003,	Caribbean Cooperation in Health initiative	Includes NCDs as a priority area
2005-2009, 2010-2015	Caribbean Cooperation in Health initiative	Emphasized NCDs as a priority area
2001	CARICOM Heads of Government, Nassau Declaration	Identified NCDs as a regional priority and gave a regional approach to NCDs
2005	Caribbean Commission on Health and Development	Identifies NCDs as a super priority
2007 Sep	CARICOM Heads of Government Summit on NCDs	*NCD Summit Declaration “Uniting to Stop the Epidemic of Chronic NCDs” or the “Port of Spain Declaration”*
2007 Oct	CARICOM Agricultural Ministers Meeting	Discussed impact of food and agricultural policies on NCDs and made 13 commitments to combat NCDs
2008 Sep	CARICOM Heads of Government	Celebrated the first Caribbean Wellness Day on 13 September with support of PAHO
2008 Nov	Anniversary of CARICOM Summit CARICOM’s Council for Human and Social Development (COHSOD)	Identified the connection between health and education and noted the importance of physical activity and healthy school meals
2009 June	Meeting of CARICOM’s Council for Human and Social Development (COHSOD)	Agreed that achieving MDGs deadline would result in reduced rates of NCDs
2009 July	CARICOM Heads of Government	Endorsed the Caribbean Wellness Day slogan of “Love That Body.”
2009 Nov	Meeting of 10 CARICOM countries, CARICOM Secretariat’s Health Desk, experts from PAHO and University of the West Indies	Reviewed the “Strategic Plan of Action for the Prevention and Control of NCDs in the Countries of the Caribbean Community”
2010	CARICOM’s Council for Human and Social Development (COHSOD)	Endorsed the “Strategic Plan of Action for the Prevention and Control of NCDs in the Countries of the Caribbean Community”
2010 Sep	Regional NCD meeting at Trinidad and Tobago with support from PAHO and Inter- American Development Bank	The NCD focal points and Chief Medical Officers reviewed and evaluated the compliance with the commitments of the declaration and shared plans to advance agenda nationally and regionally
2011 Dec	Regional NCD meeting at Trinidad and Tobago with support from PAHO and Inter- American Development Bank	Reviewed and evaluated the compliance with the commitments of the declaration and shared plans to advance agenda nationally and regionally

**Table 2:** Milestones of “Port of Spain Declaration” from CARICOM summit to UN and WHO

**Year**	**Regional/ International Meet (Venue)**	**Main Outcome**
2007 Sept	CARICOM Heads of Government Summit on NCDs (Port of Spain)	NCD Summit Declaration “Uniting to Stop the Epidemic of Chronic NCDs”
2009 April	The summit of the Americas (Port of Spain)	CARICOM members reaffirmed WHO/PAHO and CARICOM policies for NCD prevention and control
2009 July	Conference of CARICOM Heads of Government (Guyana)	Advocate for UN General Assembly Special Session on NCDs
2009 Nov	Commonwealth Heads of Government meeting (Port of Spain)	12 CARICOM members emphasized the commitment for NCDs and support for a UN meeting on NCDs to be held in 2011 under the auspices of UN General Assembly
2010 Feb	CARICOM, Brazil, and the WHO (New York)	Advocated for UN HLM on NCDs before UN permanent representatives in New York
2010 May	The proposal by CARICOM to UNGA (New York)	UNGA agreed to an HLM on NCDs in Sep 2011
2010 July	Conference of CARICOM Heads of Government (Jamaica, Montego Bay)	UN- Secretary-General Ban Ki-moon pledged support for UN-HLM on NCDs
2010 Sep	Second CARICOM- Japan Ministerial Conference (Tokyo)	NCDs were included in the UN Millennium Development Goals (MDGs) Summit
2010 Nov	Summit of Group of Twenty-G20 (Seoul)	The problems posed by NCDs were brought up
2010 Nov	Asia Pacific Economic Cooperation (Yokohama)	Leaders agreed on the necessity to enhance “NCD” control
2010 Dec	Discussions by CARICOM members for UN-HLM	Scope and modalities of HLM were approved- 2 days meeting 19-20 September 2011
2011 Sep	UN High-Level Meeting with 113 member states (New York)	United Nations Political Declaration on the Prevention and Control of NCDs (resolution A/RES/66/2)
2013 May	World Health Assembly- 66^th^ (Geneva)	WHO Global Action Plan for the Prevention and Control of NCDs 2013-2020 (resolution WHA66.10
